# A Cross-Sectional Study on Awareness and Knowledge of Sleeve Gastrectomy in the Eastern Province of Saudi Arabia

**DOI:** 10.7759/cureus.49428

**Published:** 2023-11-26

**Authors:** Mohammed A Buhalim, Mashael A Alhussain, Ibraheem A Alhusain, Omar M Aldaej, Yaqin A AlAli, Abdulrahman K Aldrweesh, Munif M Alshammari

**Affiliations:** 1 General Surgery, King Faisal University, Hofuf, SAU; 2 General Practice, King Faisal University, Hofuf, SAU; 3 Neurosurgery, King Saud Medical City, Riyadh, SAU

**Keywords:** health education, public health, complications, sleeve gastrectomy, bariatric surgery, obesity

## Abstract

Background: Obesity is a global health concern associated with a plethora of chronic diseases. Genetic and lifestyle factors play a pivotal role in its development, making it a significant challenge for healthcare systems worldwide. In this context, sleeve gastrectomy has emerged as a prominent bariatric surgical intervention, but the level of awareness and knowledge regarding its indications and complications in the Eastern Province of Saudi Arabia is underexplored.

Methods: This descriptive cross-sectional study was conducted over one year, from January 2022 to December 2022. The study aimed to assess the level of awareness among adults residing in the Eastern Province of Saudi Arabia regarding the indications and potential complications associated with sleeve gastrectomy. The study utilized random sampling and distributed a well-designed questionnaire in both English and Arabic. The questionnaire, validated for reliability, covered demographic data, general knowledge of sleeve gastrectomy, awareness of its indications, and knowledge of potential complications.

Results: The study included 1730 participants, with a majority in the 18-25 age group, predominantly female, and possessing a bachelor's degree. Awareness of sleeve gastrectomy was remarkably high, with 99% of respondents having heard of it, but only 50.1% accurately recognized the correct body mass index range for classifying obesity. Knowledge scores revealed 61.7% with poor knowledge, 31% with moderate knowledge, and 7.2% with good knowledge levels. Only 56.1% correctly identified the indications for sleeve gastrectomy. While participants displayed awareness of common complications, such as nutritional deficiencies, knowledge gaps persisted.

Conclusion: This study exposes significant gaps in awareness and knowledge regarding sleeve gastrectomy, particularly concerning its indications and potential complications. It underscores the urgent need for targeted educational initiatives and active healthcare provider engagement in disseminating accurate information. Bridging these knowledge gaps through tailored public health campaigns can empower individuals to make informed decisions about the management of obesity.

## Introduction

Obesity is a global health issue profoundly affecting individuals' daily lives and contributing to the onset of numerous chronic diseases [1]. Studies underscore the substantial influence of genetic factors and personal behaviors in the development of obesity [1,2]. Its strong association with prevalent health conditions such as hypertension, type 2 diabetes, and a spectrum of other pathologies has elevated it to the status of a paramount global health concern [2].

The prevalence of obesity continues to surge, with higher epidemic proportions evident in both developed and developing nations [3]. As of 2016, approximately 13% of the global adult population, comprising 11% of men and 15% of women, grappled with obesity [3]. Recently, Saudi Arabia has also witnessed a surge in obesity rates, impacting both males and females, with the latter experiencing a higher incidence [4].

Addressing obesity is a multifaceted challenge, necessitating a step-by-step approach. Initial interventions typically commence with lifestyle adjustments and behavioral therapy. In addition to these lifestyle modifications, pharmacological interventions constitute the second line of management. Bariatric surgery, as the third-line treatment, is increasingly recognized as one of the most effective and sustainable interventions, particularly for severely or moderately obese patients with comorbid conditions unresponsive to non-surgical approaches [5].

Bariatric surgery encompasses several surgical options, with sleeve gastrectomy being the most widely practiced technique [5]. Nonetheless, it is crucial to recognize that sleeve gastrectomy, like any surgical procedure, presents a range of potential complications, some of which can lead to life-threatening situations [6]. Postoperative complications vary in prevalence, encompassing major issues such as hemorrhage and anastomotic leaks, alongside other complications like surgical site infections, abscesses, and nutritional deficiencies [6].

A previous study investigating the long-term nutritional deficiencies following sleeve gastrectomy, spanning six years, involved 209 patients [7]. The study highlighted the prevalence of nutritional deficiencies preoperatively, with 17.2% of patients experiencing anemia. Nutrient deficiencies, including iron, ferritin, folic acid, vitamin B12, magnesium, and phosphorus, were observed both pre- and postoperatively.

In the Eastern Province of Saudi Arabia, studies exploring the awareness of indications and complications associated with sleeve gastrectomy are scarce. Consequently, the present study seeks to address this research gap by gauging the perception and knowledge levels within the local community regarding sleeve gastrectomy as a treatment modality for obesity.

## Materials and methods

Study design and setting

This descriptive cross-sectional design was conducted over a one-year period from January 2022 to December 2022. The primary aim of this study was to evaluate the level of awareness among adults residing in the Eastern Province of Saudi Arabia regarding the indications and potential complications associated with sleeve gastrectomy.

Study participants

The study included adult residents of the Eastern Province, with exclusion criteria to exclude individuals under the age of 18, those residing outside the region, and participants who did not complete the questionnaire. Participants were selected through random sampling, and the questionnaire was widely disseminated through various social media platforms.

Study instrument

A well-designed questionnaire was created, available in both English and Arabic. The survey questionnaire was developed after a careful review of the literature. The questionnaire was validated by face validity and reviewed by experts in the field of family medicine and bariatric surgeries. A pilot study was conducted with 30 participants, and Cronbach's alpha showed good reliability.

The questionnaire consisted of four distinct sections, each strategically constructed to assess participants' awareness of sleeve gastrectomy. The first section gathered general demographic data, including gender, age, and educational background. The second section evaluated participants' knowledge of sleeve gastrectomy, probing and assessing their understanding of sleeve gastrectomy and the importance of body mass index (BMI). Knowledge assessment was conducted using a 19-item questionnaire. The third section assessed participants' awareness of the indications for sleeve gastrectomy, while the final section evaluated participants' awareness of potential complications associated with sleeve gastrectomy.

Data analysis

For data analysis, we used the IBM SPSS Statistics for Windows, Version 26 (Released 2019; IBM Corp., Armonk, New York, United States). Categorical variables were represented numerically, while continuous variables were summarized with the mean and standard deviation. To assess associations, we applied the chi-square test, with statistical significance set at an alpha level of 0.05. To account for the non-normal distribution of knowledge scores, as confirmed by the Shapiro-Wilk and Kolmogorov-Smirnov tests, differences in knowledge scores based on demographic characteristics were examined using the Mann-Whitney U test.

Ethical consideration

This research study received ethical approval from the Research Ethics Committee of King Faisal University (KFU-REC-2022-JAN-EA000379). Participant confidentiality was maintained, and informed consent was obtained from all participants before their involvement in the study.

## Results

Participant demographics

The study included a total of 1730 participants from the Eastern Province. As shown in Table 1, the largest age group was individuals between 18 and 25 years old, constituting nearly 60% of the respondents. Notably, the majority of participants were female, accounting for a significant proportion of the sample. Additionally, a substantial portion of the participants (62.6%) held bachelor's degrees.

**Table 1:** Sociodemographic characteristics of participants BMI: Body mass index

Variable		Frequency	Percentage
Age group	18-25 years	752	43.5
	26-35 years	370	21.4
	36-45 years	342	19.8
	46-55 years	197	11.4
	>55 years	69	4.0
Gender	Male	713	41.2
	Female	1017	58.8
Educational level	Elementary school	11	0.60
	Intermediate school	40	2.3
	High school	365	21.1
	Diploma	159	09.2
	Bachelor	1083	62.6
	Master’s degree	46	2.7
	PhD	26	1.5
Heard of sleeve gastrectomy	Yes	1712	99.0
	No	18	1.0
Heard of BMI	Yes	1439	83.2
	No	291	16.8
What is the BMI range in which we can say that this person is obese?	<18.5 (kg/m^2^)	34	2.0
	18.5-24.9 (kg/m^2^)	81	4.7
	25-29.9 (kg/m^2^)	237	13.7
	≥30 (kg/m^2^)	866	50.1
	I don't know	512	29.6

General knowledge of sleeve gastrectomy

Awareness of sleeve gastrectomy was exceptionally high among the study population, with 99% of respondents having heard about it. Furthermore, 83.2% of participants demonstrated awareness of the BMI. Half of the respondents (50.1%) accurately recognized the correct BMI range for classifying an individual as obese, which is 30 kg/m² or more.

Awareness of indications and complications of sleeve gastrectomy

The average knowledge score across the sample was 4.68 ± 3.09, with 61.7% categorized as having poor knowledge, 31% with moderate knowledge, and 7.2% demonstrating good knowledge levels. Detailed insights into the participants' knowledge regarding the indications and complications of sleeve gastrectomy are presented in Table 2. Only 56.1% of respondents correctly identified the indications for sleeve gastrectomy. These included cases related to adults with a BMI >30 who had poorly controlled type 2 diabetes and an increased risk of cardiovascular disease, adults with a BMI >40, and adults with a BMI >35 who had severe comorbidities.

**Table 2:** Assessment of participants’ knowledge regarding the indications and complications of sleeve gastrectomy BMI: Body mass index

Statement		Frequency	Percentage
Knowledge about the indications	Adults with BMI >40	913	52.8
	Adults with BMI >35 and severe comorbidities	789	45.6
	Adults with BMI >30, poorly controlled type 2 diabetes, increased cardiovascular risk	970	56.1
	For cosmetics	322	18.6
	Adults with a BMI of 18.6-24.9	83	4.8
	Adult with a BMI of more than 18.5	45	2.6
	Adults with a BMI less than 18.5	14	0.80
	I don’t know	226	13.1
Knowledge about the complication	Other nutritional and mineral deficiencies	1072	62.0
	Iron deficiency	773	44.7
	Anemia	754	43.6
	A leak of gastric content	715	41.3
	Hemorrhage	603	34.9
	Weight gain	533	30.8
	Twist of stomach	345	19.9
	Abscess	314	18.2
	Neuropathies	163	9.4
	Pulmonary embolism	158	9.1
	I don’t know	323	18.7
Level of knowledge	Poor	1068	61.7
	Moderate	537	31.0
	Good	125	7.2

Participants displayed awareness of the most common complications associated with sleeve gastrectomy, with 62% recognizing other nutritional and mineral deficiencies as a frequent issue. This was followed by iron deficiency (44.7%), anemia (43.6%), leakage of gastric content (41.3%), and hemorrhage (34.9%).

Differences in knowledge scores

The knowledge scores were further analyzed with respect to participants' sociodemographic characteristics, as presented in Table 3. The findings indicated that higher knowledge scores were significantly associated with being in the younger age group (p<0.01), female gender (p<0.01), a higher level of education (p<0.01), and understanding the correct BMI range for classifying an individual as obese (p<0.01).

**Table 3:** Differences in the score of knowledge in relation to the sociodemographic characteristics of participants BMI: Body mass index

Variable		Knowledge score	p-value
Age group	≤35 years	4.98 ± 3.23	<0.01
	>35 years	4.14 ± 2.76	
Gender	Male	4.22 ± 3.04	<0.01
	Female	5.01 ± 3.09	
Educational level	Diploma or below	4.18 ± 2.86	<0.01
	Bachelor or higher	4.94 ± 3.18	
Heard of BMI	Yes	5.08 ± 3.08	<0.01
	No	2.73 ± 2.33	
Knowledge about the BMI range of obesity	<30 (kg/m^2^)	4.92 ± 2.96	<0.01
	≥30 (kg/m^2^)	5.69 ± 2.95	

## Discussion

The current study offers valuable insights into the level of awareness within the general population regarding the indications and complications of sleeve gastrectomy in the Eastern Province of Saudi Arabia. The study's evaluation of the population's knowledge concerning the indications and complications of sleeve gastrectomy revealed significant gaps in awareness. Notably, 61.7% of participants were categorized as having poor knowledge. These findings are consistent with previous research in the field, which has observed a lack of awareness among patients and the general population regarding the indications and complications of sleeve gastrectomy. The scarcity of proper education within the community is a plausible explanation for these findings, aligning with previous reports [8-10]. Proper health education and awareness campaigns targeting the public are imperative to ensure patients receive the most appropriate treatment for their specific needs [11,12].

The study identifies several significant factors that influence knowledge levels among the population. Younger age, female gender, higher education levels, awareness of gastric sleeves, and knowledge of the correct BMI range for classifying an obese person are associated with higher knowledge scores. These findings are consistent with previous research, highlighting the pivotal role of education and awareness in shaping understanding [13,14]. It is evident that appropriate health education, especially among high-risk populations, can significantly enhance awareness and knowledge levels.

The population's knowledge regarding the indications for sleeve gastrectomy appears to be relatively fair. The study reports an 83.2% awareness rate of BMI among participants. Understanding the role of BMI in assessing obesity is fundamental to informed decision-making about bariatric surgery. While these results demonstrate a reasonable level of understanding regarding the indications for sleeve gastrectomy, there is still room for improvement. Educational initiatives that specifically target these areas of knowledge can further enhance awareness of the criteria for undergoing the procedure.

Conversely, the population's knowledge of potential complications following sleeve gastrectomy did not reach the desired level. Awareness of some of the most common complications, such as iron deficiency, anemia, leakage of gastric content, and hemorrhage, was suboptimal. Addressing this knowledge gap is essential to ensure patients are adequately informed about the potential risks and benefits of the procedure [15,16].

The divergence in knowledge levels among different populations underscores the significance of tailored educational campaigns and the need for healthcare providers to be actively involved in patient education. For instance, in a study conducted by Almulhem and colleagues [17], different complications were reported by participants, indicating varied perceptions and expectations regarding sleeve gastrectomy. These discrepancies emphasize the importance of dispelling misconceptions and ensuring that patients have accurate information about the procedure.

It is essential to acknowledge the study's limitations. While the sample size is substantial, the data collected are specific to the Eastern Province of Saudi Arabia. Regional differences in awareness and knowledge levels may exist. Additionally, the study is cross-sectional, offering a snapshot of awareness at a particular point in time. Therefore, longitudinal studies may be needed to assess the long-term impact of awareness campaigns.

## Conclusions

In conclusion, this study has unveiled essential insights into the awareness of the general population in the Eastern Province of Saudi Arabia regarding sleeve gastrectomy, specifically focusing on its indications and potential complications. While the high awareness of the procedure is a positive starting point, the study has highlighted significant knowledge gaps that necessitate immediate attention. Suboptimal knowledge levels regarding the procedure's criteria and possible postoperative complications underscore the urgency of targeted educational initiatives and active engagement by healthcare providers. By bridging these knowledge gaps, tailored public health campaigns and well-informed healthcare discussions can empower individuals to make informed decisions about the management of obesity. Ultimately, enhanced awareness and understanding can contribute to more effective and patient-centered healthcare in the ongoing battle against obesity and its associated health concerns.
